# The anisotropy in the optical constants of quartz crystals for soft X-rays

**DOI:** 10.1107/S1600576720016325

**Published:** 2021-02-19

**Authors:** A. Andrle, P. Hönicke, J. Vinson, R. Quintanilha, Q. Saadeh, S. Heidenreich, F. Scholze, V. Soltwisch

**Affiliations:** a Physikalisch-Technische Bundesanstalt (PTB), Berlin, Germany; b National Institute of Standards and Technology (NIST), Gaithersburg, Maryland, USA; c ASML Netherlands BV (ASML), The Netherlands

**Keywords:** optical constants, quartz, anisotropy, soft X-ray reflectometry

## Abstract

The refractive index of a *y*-cut SiO_2_ crystal surface is reconstructed from orientation-dependent soft X-ray reflectometry measurements in the energy range from 45 to 620 eV. Owing to the anisotropy of the crystal structure in the (100) and (001) directions, a significant deviation of the measured reflectance at the Si *L*_2,3_ and O *K* absorption edges is observed. The anisotropy in the optical constants reconstructed from these data is also confirmed by *ab initio* Bethe–Salpeter equation calculations for the O *K* edge. This new experimental data set expands the existing literature data for quartz crystal optical constants significantly, particularly in the near-edge regions.

## Abstract 2

The refractive index of a *y*-cut SiO_2_ crystal surface is reconstructed from polarization-dependent soft X-ray reflectometry measurements in the energy range from 45 to 620 eV. The reconstructed anisotropy in the optical constants is also confirmed by *ab initio* Bethe–Salpeter equation calculations of the O *K* edge.

## 1. Introduction   

Silicon dioxide (SiO_2_) is very well known for its polymorphism (Brückner, 1970 ▸). One of its crystalline forms is called quartz and is classified into different types (I–IV) depending on the manufacturing process and the resulting impurities (Brückner, 1970 ▸; Kitamura *et al.*, 2007 ▸). Quartz glass is used today in a wide variety of applications, from simple laboratory glassware and optics to semiconductor manufacturing and lithography photomasks. In the field of optoelectronics and micro-electro-mechanical systems (MEMS), quartz, similar to pure silicon because of its physical properties, is often used as a carrier material for various mirrors, nanostructures and other functional surface features.

For the development of new optical components with tailor-made properties – *e.g.* maximum reflectivity in a certain wavelength range for a mirror – reliable material knowledge is required. The determination of accurate optical constants is a key factor for the modeling of light–matter interactions and for the design of novel optical devices. In the literature many different measurements of the optical constants of amorphous SiO_2_ exist (Yanagihara *et al.*, 1988 ▸; Tripathi *et al.*, 2002 ▸; Filatova *et al.*, 1996 ▸, 1999 ▸). A good overview of measurements for amorphous silica glass from EUV to infrared is given by Kitamura *et al.* (2007 ▸).

For certain materials, however, the optical constant or dielectric permittivity is dependent not only on the wavelength but also on the wavevector. This dependence is also called spatial dispersion or optical anisotropy and can occur, for example, in a perfect cubic crystal that should be completely isotropic. The effect of optical anisotropies near the absorption edges was theoretically postulated by Ginzburg (1958 ▸). In the vicinity of a strong absorption edge, the dielectric function and thus the refractive index increases strongly. The wavelength within the medium can therefore shrink to the order of the lattice constant. These anisotropies have been observed in various materials (Yu & Cardona, 1971 ▸; Pastrnak & Vedam, 1971 ▸; Letz *et al.*, 2003 ▸) in the past and have also been confirmed for quartz at the O *K* edge by X-ray absorption near-edge spectroscopy (Taillefumier *et al.*, 2002 ▸).

The primary interactions in the low-energy range of X-rays are photoabsorption and coherent scattering. These interactions can be described well with the complex atomic scattering factors *f*(0) (the Fourier transformation of the charge distribution) assuming that the individual atoms scatter independently. The total coherent scattering intensity can then be described as the sum of the scattering amplitudes of the individual atoms. For long wavelengths, compared with atomic dimensions and scattering amplitudes which are in phase, the angular dependence of the scattering factor disappears. The interaction of X-rays and matter is then often described by optical constants such as the complex refractive index 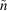 or dielectric constant ε^1/2^ (for non magnetic materials): 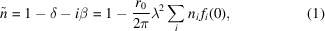
where *r*_0_ is the classical electron radius, λ is the photon wavelength, *n*_*i*_ is the atom number density and *f*(0) = *f*_1_ + *if*_2_ represents the complex atomic form factor. The real part 1 − δ and the imaginary part β of the complex refractive index are often referred to as optical constants or the n&k values of a certain material. This approximation is sufficient for photon energies above 30 eV and far away from the absorption edges (Henke *et al.*, 1993 ▸). To experimentally verify or determine these optical constants, the reflection, the transmission or the absorption of the material is measured at different wavelengths (Poelman & Smet, 2003 ▸). However, transmission experiments in the soft X-ray range usually require very thin and free-standing material samples, which are not always available. Therefore, indirect methods (total electron or fluorescence yield) are often used in this spectral range to determine the absorption. The real part of the refractive index can then be calculated using the Kramers–Kronig relationship (Filatova & Lukyanov, 2002 ▸; Soufli & Gullikson, 1997 ▸). A widely used alternative is the measurement of the specular reflectivity. This is known as X-ray reflectometry (XRR) (Stoev & Sakurai, 1997 ▸) and is mostly used to determine the thickness of thin films or multilayer systems with sub-nanometre precision (Gil & Windover, 2012 ▸).

In this paper we evaluate orientation-dependent reflection measurements with soft X-rays on a quartz crystal. Our data cover a photon energy range from 45 to 620 eV and include two crystal orientations. To limit the numerical effort of the global optimization of optical constants in a large energy range, an adapted meta-heuristic optimization algorithm (differential evolution; Storn & Price, 1997 ▸) was combined with a quasi-Newton method (Byrd *et al.*, 1995 ▸). The optical constants at the absorption edges reconstructed from the soft X-ray reflectivity measurements confirm the theoretically expected anisotropy of quartz in the complex refractive index. Furthermore, an unexpected anisotropy in front of the Si *L*_2,3_ edge can be observed. The observed anisotropy at the O *K* edge is also confirmed by *ab initio* simulations using *OCEAN* (obtaining core excitations from *ab initio* electronic structure and NIST BSE) calculations (Gilmore *et al.*, 2015 ▸; Vinson *et al.*, 2011 ▸).

## 2. Experimental details   

Soft X-ray reflectometry measurements were conducted at the X-ray radiometry beamline, operated by the Physikalisch-Technische Bundesanstalt (PTB), at the electron storage ring BESSY II in Berlin (Scholze *et al.*, 2001 ▸). The beamline covers the photon energy range from 45 to 1800 eV and is designed to produce a beam with low divergence (<1 mrad) with minimal halo.

The sample was mounted on a six-axis goniometer in the ellipso-scatterometer under ultrahigh vacuum conditions. The angle of incidence θ_i_ was aligned with respect to the beam with an uncertainty of 0.01°. The angle of incidence was varied between 0 and 88.7° in the *s*-polarization direction from the sample normal. The measured range of incident angles was adapted to the different photon energies in order to effectively cover the relevant angle range. The analyzed quartz (type II) surface (*y* cut) was polished (roughness average Ra < 1 nm) and aligned perpendicular to the plane of incidence. The measured direction was set with an azimuthal angle of 0 ± 0.08° for the (001) direction and 90° for the (100) direction. The specular reflection was measured with a GaAsP photodiode mounted on a movable detector arm inside the vacuum chamber and normalized to the incoming photon flux. In addition, to resolve the anisotropy of the crystal structure of quartz, the reflection measurements in the near-edge region of Si *L*_2,3_ and O *K* were performed with a energy increment slightly lower than the energy resolution of the beamline (Scholze *et al.*, 2001 ▸) (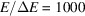). The total photon energy range covered for the quartz measurements is between 45 and 620 eV.

**Figure 1:**
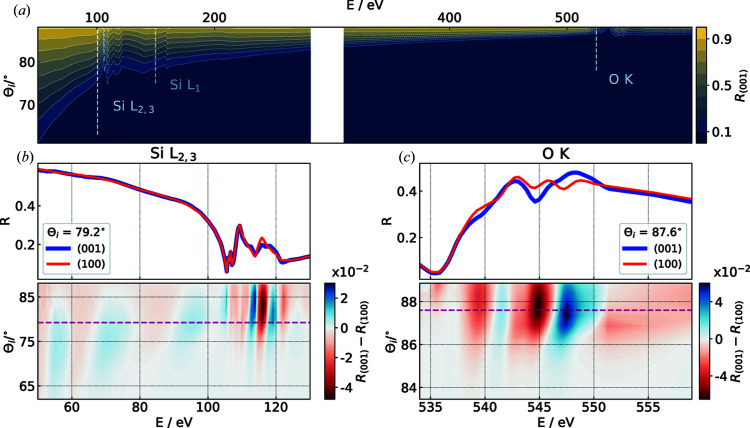
(*a*) Specular reflectance map of quartz (*y* cut) for the measured θ_i_ and photon energy range *E* in the (001) direction. The white region marks the area not measurable owing to the carbon edge. (*b*), (*c*) Comparison of the measured reflections for the crystal directions (100) and (001) at a fixed angle of incidence θ_i_ around the (*b*) Si *L*_2,3_ and (*c*) O *K* absorption edges. The lower maps in (*b*) and (*c*) show the respective anisotropy maps.

In Fig. 1 ▸(*a*) the measured specular intensity as a function of θ_i_ and *E* is shown as a contour map for the (001) direction. Figs. 1 ▸(*b*) and 1 ▸(*c*) show details of the anisotropy at the absorption edges of Si *L*_2,3_ and O *K*. The line plots at a fixed angle of incidence θ_i_ reveal that the measured anisotropy at the absorption edges is not a measurement artifact, since the measurement uncertainty is within the smallest line width. In addition, a weak birefringence below the Si *L*_2,3_ absorption edge can also be observed in the anisotropy map in Fig. 1 ▸(*b*).

## 3. Reconstruction of the optical constants from reflectivity measurements   

If the complex refractive index 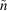 of a material is known, the expected reflectivity from the surface can be calculated as a function of the incidence angle θ_i_. The change of the wavevector component *k*_*z*_ at the *j* interface can be written as
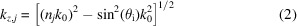
with *k*_0_ the incident wavevector. By using the Fresnel coefficients, the reflection and transmission through the medium can then be calculated directly. However, this is not sufficient to describe the measured reflectivities of the quartz crystal. The contamination of the crystal surface with carbon and water must be considered in the simulations, as the sample could not be cleaned in vacuum. The transfer-matrix method provides a method to calculate the specular reflectivity for a multilayer system depending on the layer thickness and the roughness (Gibaud, 1999 ▸). Assuming a Gaussian distribution of roughness and interdiffusion, the modified Fresnel coefficients 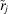 and 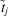 for each interface *j* can then be written (Croce & Névot, 1976 ▸) as
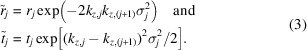
The parameter σ_*j*_ represents the mean-square intermixing at the *j*th interface.

**Figure 2:**
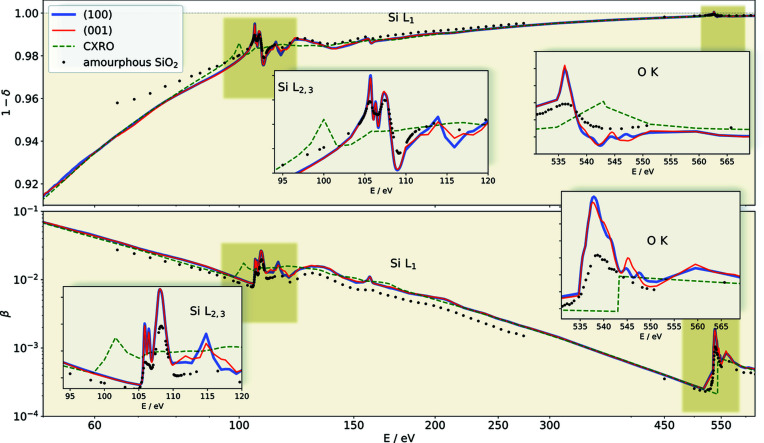
Reconstructed refractive index of a quartz crystal. Shown are the 1 − δ and β parts for the ordinary (001) (red line) and extraordinary (100) (blue line) orientations of the crystal compared with the SiO_2_ Center for X-ray Optics database values (green dashed line) (Henke *et al.*, 1993 ▸) and with the optical constants from amorphous quartz (black points) (Filatova *et al.*, 1999 ▸). The insets show a magnified view of the reconstructed anisotropy in the absorption edge areas. The data are available online (Andrle *et al.*, 2020 ▸).

To stabilize the stratified system and the results for the reconstruction of the optical constants, all measured reflectivities (2 × 10^5^) at different incidence angles θ_i_ and photon energies *E* were optimized simultaneously. Owing to the lack of suitable n&k models in the EUV spectral range, the challenge in the optimization process arises in the very large number of degrees of freedom. For each θ scan at a fixed wavelength an independent refractive index *n* had to be assumed. In the reconstruction attempts, it quickly became apparent that the use of fast gradient methods for the optimization led to inconsistent results depending on the selected start parameters. This problem of getting stuck in local minima while simultaneously optimizing layer parameters and optical constants is well known (Cao *et al.*, 1994 ▸). More suitable for the global minimization of a problem are heuristic or meta-heuristic methods. The convergence of the differential evolution method (DE) (Storn & Price, 1997 ▸), however, is very slow when several hundred degrees of freedom are considered. But the present problem can be easily split up. For a defined geometric model **p** of the layer system there is only one refractive index that can best describe the measured reflectivity. Therefore, a combination of two different optimization methods might be used. In an external optimization, the best combination of geometric parameters is evaluated by means of DE, while a quasi-Newton algorithm (L-BFGS-B; Byrd *et al.*, 1995 ▸) determines the corresponding optical constants (δ, β), for every layer, inside the objective function χ′ of DE. The objective function can then be written as
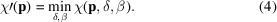
The optical constants obtained with this approach, in the energy range from 45 to 620 eV, are in good agreement with tabulated values of existing databases (Henke *et al.*, 1993 ▸; Chantler, 2000 ▸; Palik, 1998 ▸) for SiO_2_ with an expected density of 2.65 g cm^−3^. However, this perfect agreement is only valid in the regions far away from the absorption edges of silicon (Si *L*_2,3_) and oxygen (O *K*). The absorption edge positions of the tabulated data are energetically clearly shifted compared with our data, and of course the fine structure is also missing. The insets in Fig. 2 ▸ highlight the behavior of the refractive index around the absorption edges and allow us to visualize the quartz anisotropy. The orientation dependence of the fine structure can be understood by considering the local environment of the absorbing atom. The unoccupied Si *d* orbitals are split by the tetrahedral symmetry of the neighboring oxygen atoms into doubly degenerate *e* and triply degenerate *t*_2_ orbitals, with the broad absorption around 115 eV made up of transitions into *e*: 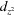 and 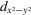. These orbitals are split locally by distortion in the oxygen tetrahedra and, through hybridization, by the long-range anisotropy of quartz. Similarly, at the O *K* edge, the features from 545 to 550 eV are due to hybridization with these same Si *d* orbitals (Wu *et al.*, 1998 ▸). Comparing the results with the optical constants for amorphous quartz (Filatova *et al.*, 1999 ▸) a difference in the fine structure can be seen. The difference is more pronounced at the oxygen absorption edge.

For this result, however, the model of a pure quartz substrate had to be revised. To compensate for the effect of the substrate’s contamination due to the adsorption of volatile organic materials as the sample was handled under normal laboratory conditions, an ultra-thin carbon-like layer with unknown optical and dimensional parameters was considered for the optimization model. It has been reported that ultra-thin organic coatings can significantly influence the coating thicknesses derived from X-ray reflectometry because of environmental contamination (Gil & Windover, 2012 ▸). At the carbon absorption edge, we cannot measure the reflectivity. Therefore, the parameters of the contamination are unknown and it is difficult to distinguish between the different effects like roughness or inter-diffusion that occur at the surface of the crystal. An equivalent problem can also be observed in the reconstruction of the optical constants.

The reconstructed surface layer thickness of ∼0.6 nm with an r.m.s. roughness of ∼0.5 nm, which is below the roughness average from the polishing, also seems reasonable for the contamination of the quartz surface. This assumption is confirmed by the following simulations of the O *K* edge, which shows that modeling errors have a significant influence on the reconstruction of the optical constants in the near-edge region. To better characterize the surface, for future studies, further experiments like X-ray fluorescence measurements are needed.

## 4. Theoretical modeling of the O *K* absorption edge of quartz   

Several groups have already published calculations of X-ray absorption fine structure at the O *K* edge (Gougoussis *et al.*, 2009 ▸; Taillefumier *et al.*, 2002 ▸). In these simulations the core–hole excited state was modeled using density functional theory (DFT). A core-level electron is removed from the absorbing atom, and the electronic system is allowed to relax fully.

Here we use instead the method of the Bethe–Salpeter equation (BSE). In this equation the ground state is modeled by DFT and the core–hole interactions of the excited state are explicitly calculated. The main difference between the two approaches is that for BSE the electronic relaxation is performed by means of a linear response to the core–hole, and for the DFT approach the exchange interaction between electron and hole is approximated.

**Figure 3:**
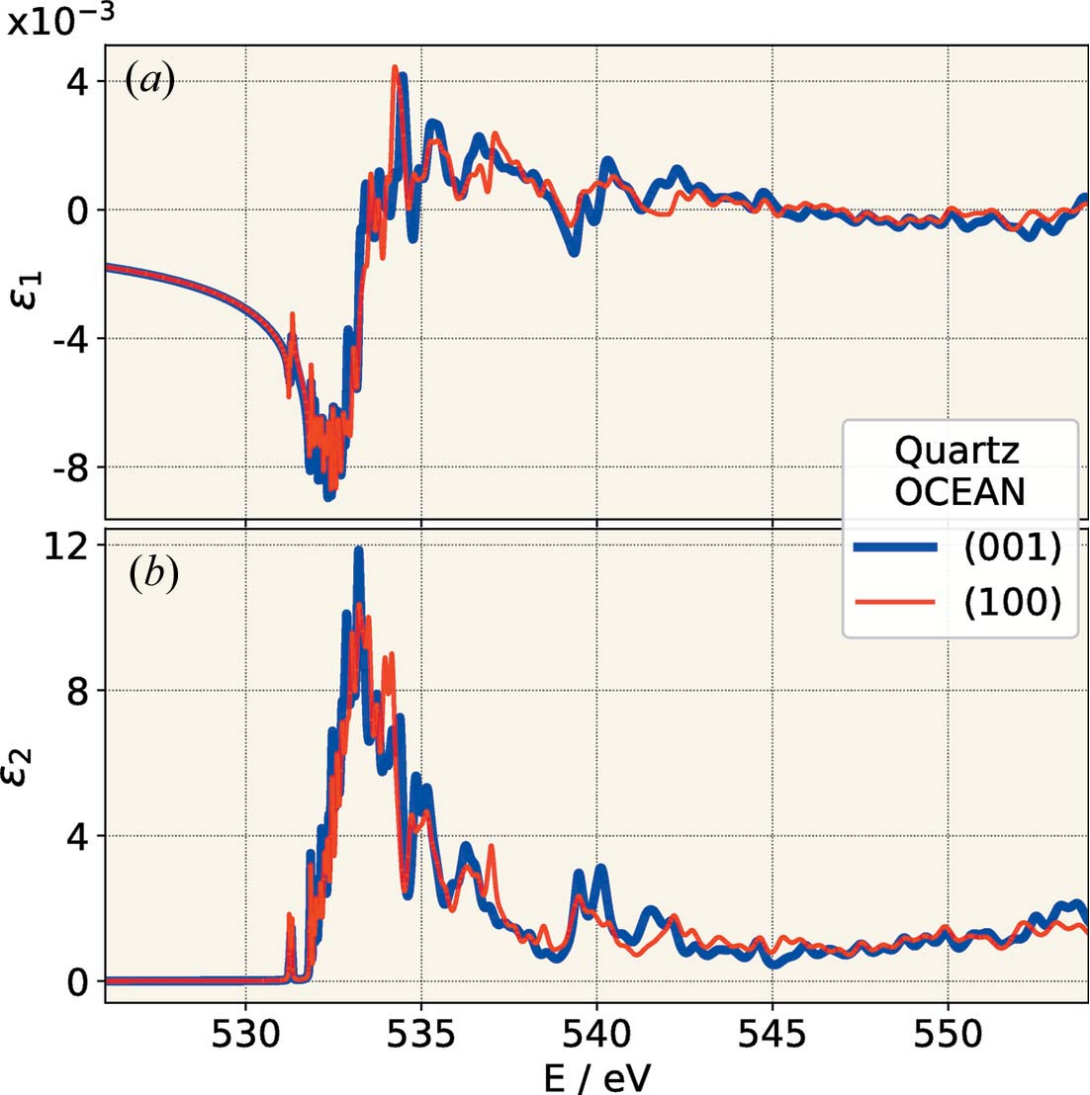
Comparison of the *OCEAN* forward calculation of the permittivity (*a*) ε_1_ and (*b*) ε_2_ at the O *K* absorption edge for the extraordinary (100) and ordinary (001) orientation of the crystal.

We performed calculations^1^ at the O *K* absorption edge using the *OCEAN* (version 2.5.2) (Gilmore *et al.*, 2015 ▸; Vinson *et al.*, 2011 ▸) code (see Fig. 3 ▸). The electronic ground state was calculated with DFT within the local density approximation using the *QuantumESPRESSO* code (Giannozzi *et al.*, 2017 ▸). For both oxygen and silicon, the pseudopotentials of the Fritz-Haber-Institut from the *QuantumESPRESSO* web site were used. We used the crystal data for quartz at room temperature reported by Kihara (1990 ▸), and *cif2cell *(Björkman, 2011 ▸) was used for conversion.

### 4.1. *OCEAN* post-processing   

**Figure 4:**
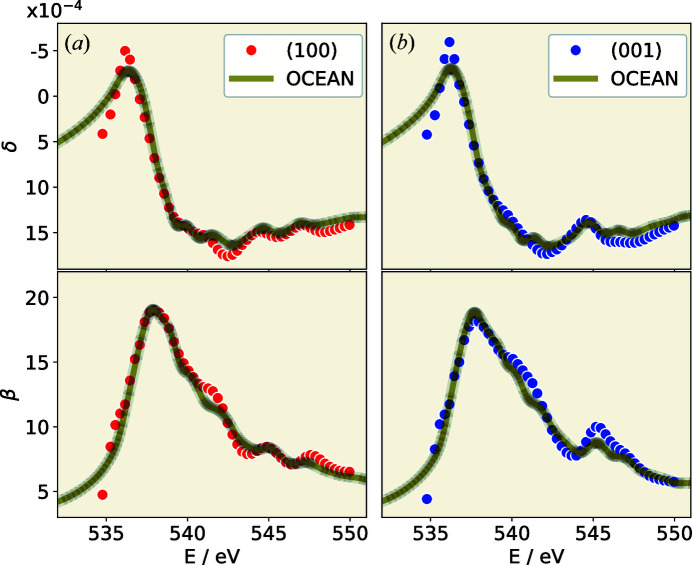
Comparison of the reconstructed optical constants (δ, β) at the O *K* absorption edge for the (*a*) extraordinary (100) and (*b*) ordinary (001) orientations of quartz with the *OCEAN* simulation of the expected behavior of a quartz crystal. For the reconstruction of the optical constants from the reflectivity measurements a contamination of the surface was modeled.

For a comparison of the calculated complex permittivity (ε)^1/2^ = (ε_1_ + *i*ε_2_)^1/2^ with the experimentally determined optical constants at the O *K* edge, several corrections must be applied to the forward calculation. Owing to the limitations of DFT, a polynomial correction of the energy scale has been applied, which essentially allows a slight modification of the position of each peak. In addition, the lifetime broadening as well as the energy resolution of the beamline must be approximated with a Voigt distribution. The contributions for energetically less bound electrons are considered by Ebel polynomials (Ebel *et al.*, 2003 ▸). The corresponding model parameters are determined by means of a least-squares optimization. Thereby both ε_1_ and ε_2_ are optimized simultaneously with an identical set of parameters. The results of the *OCEAN* post-processing for the O *K* edge are compared against the reconstructed optical constants in Fig. 4 ▸. The shape of the δ and β curves in the region of the oxygen edge is already reasonably well reproduced.

The BSE approach makes a number of approximations which limit its ability to fully reproduce experimental data. First, the atomic nuclei are treated as fixed in their equilibrium position. This neglects both the movement of the atoms in the ground state, in the form of vibrations and zero-point motion, and the exciton–phonon scattering in the excited state. For stable crystalline systems without very light nuclei (hydrogen) the effects of this approximation are minor. Secondly, BSE models X-ray excitation as a single electron–hole pair, which can be problematic for atoms with highly localized *d* or *f* electrons. Thirdly, but most importantly in this work, the conduction electron states are computed using DFT within the local density approximation. The DFT is known to underestimate excitation energies including band gaps, and the excited electron states have an infinite lifetime within the DFT. Higher-level theories, *e.g.* GW self-energy calculations, can be used to correct the DFT energies, including lifetimes. Lastly, we approximate the many-body photon absorption process as a single-electron interaction and treat the electrons as quasi-particles. These effects can be grouped into an unknown proportionality constant.

A comparison of the *OCEAN* post-processing results with the reconstructed refractive index from the reflectometry measurements at the O *K* edge is shown in Fig. 4 ▸. An atomically thin organic contamination of the quartz surface was assumed for the reflectometry model on which the n&k fit of the *OCEAN* post-processing is based. Omitting the contamination layer in the model results in a significant difference which can best be visualized by comparing the anisotropies.

### 4.2. *OCEAN* anisotropy for different reflectometry models   

**Figure 5:**
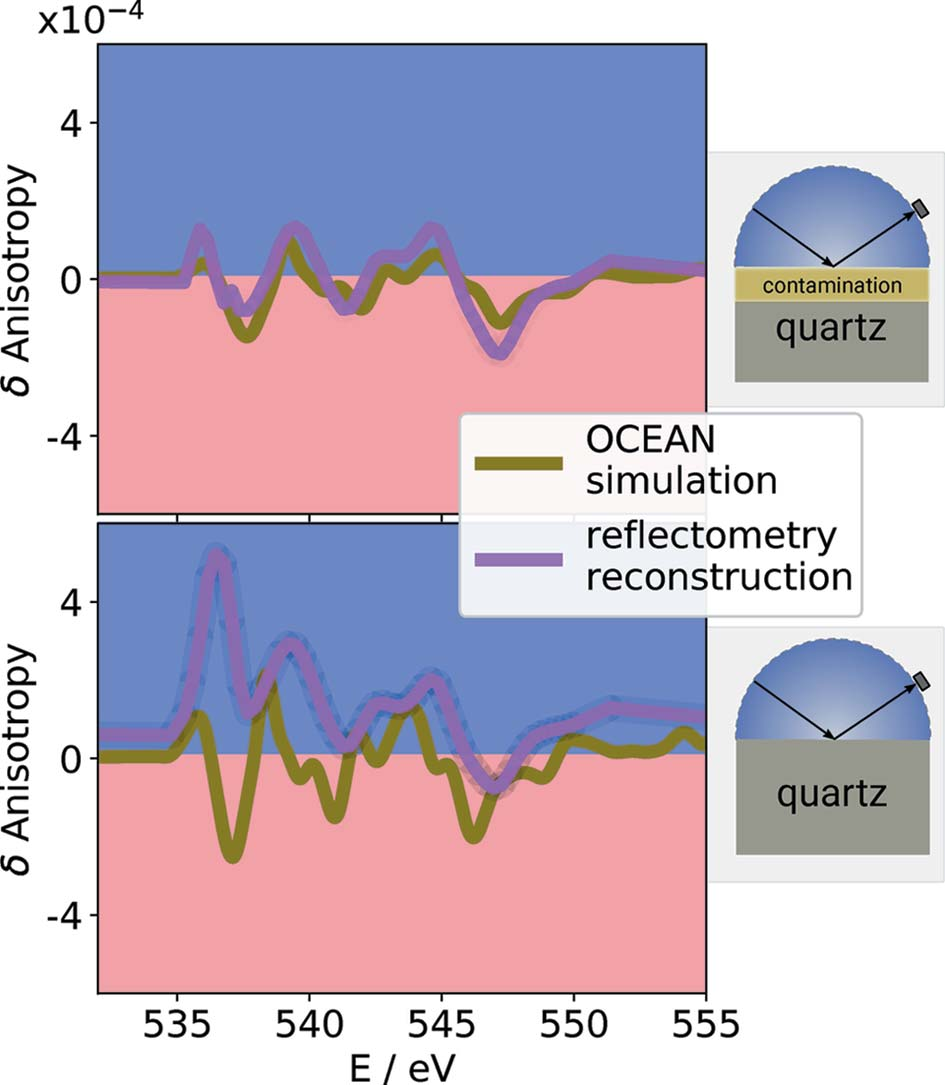
The reconstructed anisotropy at the O *K* absorption edge of the real part of the refractive index δ in comparison with the anisotropy as obtained from the *OCEAN* simulation and subsequent post-processing (see text). A reasonable agreement in anisotropy is obtained with a reflectometry model that also allows surface contamination (*a*).

To allow an unbiased comparison, the parameters of the *OCEAN* post-processing were also adjusted to n&k values determined by a reflectometry model corresponding to a pure quartz surface. The comparison of the two models is shown in Fig. 5 ▸ for the simulated anisotropies reconstructed from the measurement and adjusted on this basis. In the figure the differences for δ for both crystal orientations [(100), (001)] are shown as a function of the photon energy. In Fig. 5 ▸(*b*) the difference in δ for n&k values determined by a reflectometry model containing only the substrate and the results from the *OCEAN* post-processing to fit these n&k is displayed. The direct comparison of the two models clearly shows that the omission of the contamination layer leads to a significant difference.

Clearly, within this simplified model, the reconstruction of the optical constants in the area of the absorption edges is affected. This is also obvious from the fit results of the measured and modeled reflectivities. The anisotropy reconstructed in this way increases significantly and can no longer be correlated with the *OCEAN* simulation. This observation is on the one hand a proof for the validity of the assumed model, but at the same time it shows how crucial the model error is in the reconstruction of optical constants. First tests based on the Markov chain Monte Carlo method showed that the uncertainty for the reconstructed optical constants at the absorption edge increases. Similar observations for the uncertainties of the optical constants are reported by Soufli & Gullikson (1997 ▸) at the Si *L* edge for pure silicon. This probably correlates with the loss in reflectivity at the zero crossing of the real part of the refractive index.

## 5. Conclusions   

In this work we have determined optical constants for quartz in a broad photon energy range in the soft X-ray region and can significantly extend existing databases, especially in the vicinity of the Si *L* and O *K* absorption edges. The comparison with literature values for SiO_2_ shows a excellent agreement away from absorption edges and the expected deviations at the edges. In these spectral ranges, an anisotropy of the optical constant can be clearly verified depending on the orientation of the crystal. This work could also pave the way to identify possible stress-induced anisotropy in thin films by measuring the anisotropy near the absorption edge of the material under consideration (*e.g.* Si, O, N, C).

The measured anisotropy of quartz is also confirmed by *ab initio* BSE simulations at the O *K* edge using *OCEAN* calculations. These forward calculations confirm the model assumption of the presence of a thin surface contamination layer for the reconstruction of the optical constants. A perfect agreement between measured and calculated optical constants of quartz around the O *K* absorption edge could not yet be achieved with the present theoretical simulations. The BSE approach for the simulation requires many approximations which limit its possibilities. However, a better prediction of fine structures at the edges seems to be possible and offers potential for further chemical analysis if experimental resolution limits are reached.

## Supplementary Material

The refractive index of a y-cut SiO2 crystal surface is reconstructed from polarization-dependent soft X-ray reflectometry measurements in the energy range from 45 to 620 eV. In the data sets the 1-delta and beta values for the (100) and (001) directions of the SiO2 crystal for the different energies are provided: https://doi.org/10.5281/zenodo.4118056

## Footnotes

1 We used a plane wave energy cut-off of 55 Ry, and the isotropic dielectric constant ε_∞_ was set to 3.8. A 12 × 12 × 12 *k*-point grid and 500 bands were used for the BSE final states, while a 4 × 4 × 4 *k*-point grid and 150 bands were used for calculating the screening of the core–hole.
